# Associations of Perfluoroalkyl substances with blood lipids and Apolipoproteins in lipoprotein subspecies: the POUNDS-lost study

**DOI:** 10.1186/s12940-020-0561-8

**Published:** 2020-01-13

**Authors:** Gang Liu, Bo Zhang, Yang Hu, Jennifer Rood, Liming Liang, Lu Qi, George A. Bray, Lilian DeJonge, Brent Coull, Philippe Grandjean, Jeremy D. Furtado, Qi Sun

**Affiliations:** 1 Department of Nutrition and Food Hygiene, Hubei Key Laboratory of Food Nutrition and Safety, Ministry of Education Key Laboratory of Environment and Health, School of Public Health, Tongji Medical College, Huazhong University of Science and Technology, Wuhan, China; 2 Department of Nutrition, Harvard T.H. Chan School of Public Health, 665 Huntington Ave, Boston, MA 02115 USA; 3 Department of Biochemistry, Fukuoka University School of Medicine, Fukuoka, Japan; 4 Pennington Biomedical Research Center, LSU, Baton Rouge, LA USA; 5 Department of Epidemiology and Department of Biostatistics, Harvard T.H. Chan School of Public Health, Boston, MA USA; 6 Department of Epidemiology, School of Public Health and Tropical Medicine, Tulane University, New Orleans, LA USA; 7 Department of Nutrition and Food Studies, George Mason University, Fairfax, VA USA; 8 Department of Biostatistics, Harvard T.H. Chan School of Public Health, Boston, MA USA; 9 Institute of Public Health, University of Southern Denmark, Odense, Denmark; 10 Department of Envrionmental Health, Harvard T.H. Chan School of Public Health, Boston, MA USA; 11 Channing Division of Network Medicine, Department of Medicine, Brigham and Women’s Hospital and Harvard Medical School, Boston, MA USA

**Keywords:** Perfluoroalkyl substance, Lipid subfractions, Epidemiology

## Abstract

**Background:**

The associations of perfluoroalkyl substance (PFAS) exposure with blood lipids and lipoproteins are inconsistent, and existing studies did not account for metabolic heterogeneity of lipoprotein subspecies. This study aimed to examine the associations between plasma PFAS concentrations and lipoprotein and apolipoprotein subspecies.

**Methods:**

The study included 326 men and women from the 2-year Prevention of Obesity Using Novel Dietary Strategies (POUNDS) Lost randomized trial. Five PFASs, including perfluorooctanesulfonic acid (PFOS), perfluorooctanoic acid (PFOA), perfluorohexanesulfonic acid (PFHxS), perfluorononanoic acid (PFNA), and perfluorodecanoic acid (PFDA), were measured in plasma at baseline. For lipoprotein and apolipoprotein subspecies, total plasma was fractionated first by apolipoprotein (apo) C-III content and then by density. Each subfraction was then measured for apoB, apoC-III, and apoE concentrations, as well as triglyceride and cholesterol contents, both at baseline and at 2 years.

**Results:**

For lipids and apolipoproteins in total plasma at baseline, elevated plasma PFAS concentrations were significantly associated with higher apoB and apoC-III concentrations, but not with total cholesterol or triglycerides. After multivariate adjustment of lifestyle factors, lipid-lowering medication use, and dietary intervention groups, PFAS concentrations were primarily associated with lipids or apolipoprotein concentrations in intermediate-to-low density lipoprotein (IDL + LDL) and high-density lipoprotein (HDL) that contain apoC-III. Comparing the highest and lowest tertiles of PFOA, the least-square means (SE) (mg/dl) were 4.16 (0.4) vs 3.47 (0.4) for apoB (*P* trend = 0.04), 2.03 (0.2) vs 1.66 (0.2) for apoC-III (*P* trend = 0.04), and 8.4 (0.8) vs 6.8 (0.8) for triglycerides (*P* trend = 0.03) in IDL + LDL fraction that contains apoC-III. For HDL that contains apoC-III, comparing the highest and lowest tertiles of PFOA, the least-square means (SE) (mg/dl) of apoC-III were 11.9 (0.7) vs 10.4 (0.7) (*P* trend = 0.01). In addition, elevated PFNA and PFDA concentrations were also significantly associated with higher concentrations of apoE in HDL that contains apoC-III *(P* trend< 0.01). Similar patterns of associations were demonstrated between baseline PFAS concentrations and lipoprotein subspecies measured at 2 years. Baseline PFAS levels were not associated with changes in lipoprotein subspecies during the intervention.

**Conclusions:**

Our results suggest that plasma PFAS concentrations are primarily associated with blood lipids and apolipoproteins in subspecies of IDL, LDL, and HDL that contain apoC-III, which are associated with elevated cardiovascular risk in epidemiological studies. Future studies of PFAS-associated cardiovascular risk should focus on lipid subfractions.

## Introduction

Per- and polyfluoroalkyl substances (PFASs) are extensively used in many industrial and consumer products including stain- and water-repellent fabrics, nonstick cookware, and food packing [1]. PFASs have structural homology with fatty acids and may interfere with lipid metabolism [2], probably through activating peroxisome proliferator-activated receptors (PPAR) [3].

Most animal studies reported decreases in lipids after high-dose administration of perfluorooctanoic acid (PFOA) and perfluorooctane sulfonate (PFOS) [1], although some human studies have shown increased blood lipids at higher PFAS exposures [1, 4–7], especially when this association was examined prospectively among individuals exposed to high levels of PFOA [8, 9]. However, this positive association has not been uniformly replicated in other populations at lower exposure levels [10, 11], and data from occupational exposures are in dispute [12].

Potential reasons for these inconsistent findings may involve differences in study designs and exposure levels, and another important concern is the metabolic and functional heterogeneity of lipoprotein subspecies [13, 14]. Thus, what is commonly referred to as low-density lipoprotein (LDL), high-density lipoprotein (HDL), and other lipid fractions constitute diverse groups of lipoprotein particles with heterogeneous biological functions, varying in cholesterol and triglyceride contents, as well as attachments of apolipoprotein (apo) C and apoE [13, 15–17]. Accumulating evidence has suggested that apoC-III acts as an independent risk factor for cardiovascular disease [18–20]. Further, the apoC-III attached to HDL could significantly attenuate the beneficial metabolic action of HDL apoE and subsequently affect lipid metabolism and cardiovascular disease risk [17]. So far, association between PFAS exposures and lipoprotein subspecies has apparently not been investigated.

To fill this knowledge gap, we examined the associations of plasma PFAS concentrations with lipoproteins and apolipoprotein subspecies in men and women participating in the Prevention of Obesity Using Novel Dietary Strategies (POUNDS) Lost trial. We specifically hypothesized that PFAS concentrations would be positively associated with lipoprotein subspecies that contain apoC-III.

## Methods

### Study population

The POUNDS Lost study (ClinicalTrials.gov number: NCT00072995) was a randomized clinical trial designed to compare the effects of four energy-reduced diets with different compositions of macronutrients (i.e., fat, protein, and carbohydrate) on weight loss. The trial was conducted at two sites: the Harvard T.H. Chan School of Public Health and Brigham and Women’s Hospital, Boston; and the Pennington Biomedical Research Center of the Louisiana State University System, Baton Rouge, from October 2004 through December 2007. The details have been described previously [21]. Briefly, 811 overweight and obese men and women aged 30–70 years were randomly assigned to one of four healthy diets that followed the American Heart Association recommendations for cardiovascular health at baseline, and 645 participants (80%) completed the trial at 2 years [21]. The main finding of this trial was that weight changes were not significantly different between diet groups [21]. Of these participants, 406 were randomly selected for analysis of lipoprotein subspecies at baseline and 2 years. The current analysis finally included 326 participants who had data on both PFASs and lipoprotein subspecies. The protocol was approved by the Institutional Review Board at Harvard T.H. Chan School of Public Health, Brigham and Women’s Hospital, and the Pennington Biomedical Research Center of the Louisiana State University System, as well as by a data and safety monitoring board appointed by the National Heart, Lung, and Blood Institute. All participants provided written informed consent.

### Laboratory measurements of PFAS

Plasma concentrations of PFOS, PFOA, perfluorohexanesulfonic acid (PFHxS), perfluorononanoic acid (PFNA), and perfluorodecanoic acid (PFDA) were measured by a sensitive and reliable method based on online solid phase extraction and liquid chromatography coupled to a triple quadrupole mass spectrometer [22], with minor modifications. The concentrations of the five PFASs were all above the limit of detection (0.05 ng/mL), and the inter- and intra-assay coefficients of variation (CV) were both < 10%. PFAS concentrations in our study population were comparable to concentrations in the general US population in 2003–2004 [23].

### Laboratory measurements of lipoprotein and lipoprotein subspecies

The methodology of lipoprotein subspecies quantification has been described previously [24]. Briefly, plasma was thawed and incubated overnight at 4 °C in anti-apoC-III immuno-affinity columns to bind lipoproteins containing apoC-III. The unbound plasma fraction (CIII-) was eluted with phosphate-buffered saline and the bound lipoproteins (CIII+) were eluted with 3 M sodium thiocyanate. Very-low density lipoprotein (VLDL) was isolated from each fraction by ultracentrifugation at 4 °C and 25,000 rpm for 16 h. The combined intermediate-density lipoprotein (IDL) and low-density lipoprotein (LDL) fraction was then isolated following density adjustment with potassium bromide to d = 1.063 g/mL by ultracentrifugation at 4 °C and 25,000 rpm for 24 h. The remaining solution contained the HDL and other components of plasma. Therefore, six lipoprotein subspecies were generated: VLDL that contains or lacks apoC-III, IDL + LDL that contains or lacks apoC-III, and HDL that contains or lacks apoC-III. Among these subspecies, apoB, apoC-III, and apoE concentrations were further assayed using sandwich ELISAs (Academy Biomedical, Houston, TX), and cholesterol and triglyceride concentrations were determined using enzymatic assays (Thermo Electron Corp, Waltham, MA). The samples from the same participants were assayed in the same run by the same technicians in a random sequence, and any sample with an intra-assay CV > 15% was repeated.

### Assessments of covariates

Using standardized questionnaires, we obtained information on age, sex, race, educational attainment, smoking status, and alcohol consumption [25]. Physical activity was estimated using the Baecke physical activity questionnaire, which included 16 items inquiring about levels of habitual physical activities [26]. Body weight and waist circumference were measured at baseline, 6, 12, 18, and 24 months. Body mass index (BMI) was calculated as body weight in kilograms divided by height in meters squared.

### Statistical methods

The comparisons between participants included in the current analysis and those excluded were tested by the Student’s *t* test for normally distributed variables, the Wilcoxon rank-sum test for skewed variables, and the chi-square test for categorical variables. The associations between baseline PFAS and the lipoprotein and lipoprotein subspecies at baseline and at 2 years after intervention were examined using linear regression models. The least-square means and standard error (SE) of lipoprotein and lipoprotein subspecies according to tertiles of PFAS concentrations were calculated.

In terms of multivariate adjustment, we considered traditional covariates, including demographic, socio-economic, and lifestyle factors. In addition, given the clinical trial study design, we further included dietary intervention groups in the model. Lastly, considering that lipid-lowering medication use might confounder the association of interest, we also took this variable into account in multivariate analyses. Specifically, covariates considered in the multivariate models included age (years), sex (men, women), race (white, non-white), educational attainment (high school or less, some college, and college graduate or beyond), smoking status (never, former, and current smoker), alcohol consumption (drinks/week), physical activity (MET-hr/wk), BMI (kg/m^2^), four dietary intervention groups (categorical), and regular lipid-lowering medication use (yes or no). Tests of linear trend across increasing tertiles of PFAS were examined by assigning a median value to each tertile and treating it as a continuous variable. In addition, we modeled log-transformed (base 10) PFAS concentration as continuous variables.

Several sensitivity analyses were performed. First, the associations of PFAS with lipoprotein and lipoprotein subspecies were examined in strata defined by sex and race, and partial Spearman correlation coefficients (*r*_*s*_) were calculated to evaluate strength of associations. Among women, we further adjusted for menopausal status (yes or no) and hormone replacement therapy use (yes or no). Second, analyses were further restricted to participants without lipid-lowering medication use or to non-current smokers. A two-sided *P* < 0.05 was considered statistically significant. These statistical analyses were performed with SAS software, version 9.4 (SAS Institute Inc., Cary, North Carolina).

## Results

The baseline characteristics of participants included in the current study (*n* = 326) are shown in Table 1. The mean (SD) age of the participants was 52.7 (8.7) years, with a mean (SD) BMI of 32.3 (3.8) kg/m^2^. For IDL + LDL and HDL, the concentrations of these species that contain apoC-III were much lower than their counterparts that lack apoC-III. Additional file 1: Figure S1 shows a heatmap of correlations between lipoprotein and apolipoprotein subspecies. There were moderate to high correlations among most of the lipoprotein and apolipoprotein subspecies (*r*_*s*_ ranged from − 0.29 to 0.97, *P* < 0.001). Significant inter-correlations were observed between PFOS, PFOA, PFHxS, PFNA, and PFDA (*r*_s_ between 0.32 and 0.84). In addition, in comparison with the remaining participants not included in the current study, the participants included were slightly older (52.7 vs 49.6 years, *P* < 0.001) and more likely to be white (85.3% vs 75.3%, *P* = 0.01). There was otherwise no significant difference in their characteristics (Additional file 1: Table S1).

**Table 1:** Baseline characteristics of participants in the POUNDS Lost study

N	326
Age (years)	52.7 ± 8.7
Sex, men, %	39.0
Race, %	
White	85.3
Black	11.0
Hispanic	2.8
Other	0.9
BMI (kg/m^2^)	32.3 ± 3.8
Weight (kg)	91.7 ± 15.3
Weight (lbs)	202.2 ± 33.8
Waist circumference (cm)	103.2 ± 12.9
Education level, high school or less, %	9.8
Current smoker, yes, %	3.4
Alcohol consumption (drinks/week)	2.1 ± 2.8
Physical activity^a^	1.6 ± 0.1
Systolic blood pressure (mmHg)	119.8 ± 13.1
Diastolic blood pressure (mmHg)	75.1 ± 8.9
Glucose (mg/dl)	92.1 ± 11.3
PFOS (ng/ml)	23.5 (15.9, 38.0)
PFOA (ng/ml)	4.6 (3.3, 6.4)
PFHxS (ng/ml)	2.4 (1.6, 3.6)
PFNA (ng/ml)	1.5 (1.0, 2.3)
PFDA (ng/ml)	0.4 (0.2, 0.5)
Total plasma (mg/dl)	
Total cholesterol	188.9 (168.0, 225.3)
Triglycerides	96.9 (69.3, 140.6)
ApoB	85.8 (69.3, 110.5)
ApoE	7.7 (6.1, 9.3)
ApoC-III	13.4 (10.6, 17.1)
IDL + LDL containing apoC-III (mg/dl)	
Cholesterol	7.2 (5.2, 10.2)
Triglycerides	5.7 (3.9, 8.3)
ApoB	2.8 (1.8, 4.5)
ApoE	0.20 (0.13, 0.30)
ApoC-III	1.4 (0.9, 2.1)
IDL + LDL lacking apoC-III (mg/dl)	
Cholesterol	107.0 (86.9, 129.5)
Triglycerides	18.2 (13.2, 27.5)
ApoB	72.7 (58.0, 92.8)
ApoE	0.50 (0.35, 0.76)
HDL containing apoC-III (mg/dl)	
Cholesterol	6.1 (4.7, 8.2)
Triglycerides	2.4 (1.6, 3.7)
ApoE	4.1 (3.1, 4.8)
ApoC-III	10.7 (8.0, 13.9)
HDL lacking apoC-III (mg/dl)	
Cholesterol	54.9 (45.7, 67.4)
Triglycerides	6.3 (4.6, 9.6)
ApoE	2.2 (1.5, 3.2)

Data are mean ± SD, median (interquartile range), or percentage (%). ^a^Physical activity was estimated by the Baecke Questionnaire. *BMI* Body mass index, *TG* Triglycerides, *PFOS* Perfluorooctane sulfonate, *PFOA* Perfluorooctanoate, *PFHxS* Perfluorohexanesulfonate, *PFNA* Perfluorononanoic acid, *PFDA* Perfluorodecanoic acid

After multivariate adjustment including demographic and lifestyle factors, BMI, and dietary intervention groups, baseline PFOA concentrations were positively associated with apoB and apoC-III concentrations in plasma (Table 2). Comparing the highest vs lowest tertiles, the least-square means (SE) (mg/dl) were 95.4 (5.1) and 85.7 (5.3) for apoB (*P* trend = 0.03) and 15.5 (0.9) and 13.4 (0.9) for apoC-III (*P* trend = 0.007). Similar results were observed when PFOA concentrations were treated as continuous variables; for each unit increment of log_10_-transformed PFOA, there was an increment of 0.11 mg/dl in log_10_-transformed apoB (*P* = 0.007) and an increment of 0.15 mg/dl in log_10_-transformed apoC-III (*P* = 0.003) (Table 2). PFASs were not associated with plasma concentrations of total triglycerides or cholesterol.

**Table 2:** Least-square means of baseline lipids and apolipoproteins in total plasma according to tertile of PFAS^a^

	Total Cholesterol	Triglycerides	ApoB	ApoE	ApoC-III
PFOS (ng/ml)					
T1 (< 18.8)	180.9 ± 8.0	126.8 ± 11.6	86.6 ± 5.4	7.9 ± 0.4	13.7 ± 0.9
T2 (18.8–33.1)	189.3 ± 7.9	132.4 ± 11.4	90.3 ± 5.3	8.2 ± 0.4	15.2 ± 0.9
T3 (> 33.1)	190.7 ± 7.3	126.1 ± 10.5	94.0 ± 4.9	8.4 ± 0.4	14.8 ± 0.8
*P*_trend_	0.21	0.80	0.11	0.22	0.36
PFOA (ng/ml)					
T1 (< 3.7)	189.1 ± 7.9	111.1 ± 11.2	85.7 ± 5.3	7.9 ± 0.4	13.4 ± 0.9
T2 (3.7–5.6)	189.3 ± 7.6	137.3 ± 10.8	91.2 ± 5.0	8.2 ± 0.4	14.7 ± 0.9
T3 (> 5.6)	188.4 ± 7.7	131.8 ± 10.9	95.4 ± 5.1	8.4 ± 0.4	15.5 ± 0.9
*P*_trend_	0.67	0.06	0.03	0.12	0.007
PFHxS (ng/ml)					
T1 (< 1.8)	181.6 ± 7.8	119.4 ± 11.2	90.7 ± 5.2	8.4 ± 0.4	13.9 ± 0.9
T2 (1.8–3.1)	189.3 ± 7.6	133.6 ± 11.0	89.7 ± 5.1	8.2 ± 0.4	14.9 ± 0.9
T3 (> 3.1)	192.5 ± 7.8	130.8 ± 11.2	93.0 ± 5.2	8.1 ± 0.4	15.0 ± 0.9
*P*_trend_	0.15	0.37	0.55	0.39	0.28
PFNA (ng/ml)					
T1 (< 1.1)	185.6 ± 7.7	132.9 ± 11.1	87.6 ± 5.2	7.9 ± 0.4	14.4 ± 0.9
T2 (1.1–1.8)	186.1 ± 7.8	128.2 ± 11.2	89.8 ± 5.2	8.2 ± 0.4	14.4 ± 0.9
T3 (> 1.8)	190.8 ± 7.5	123.6 ± 10.8	95.0 ± 5.0	8.4 ± 0.4	15.0 ± 0.9
*P*_trend_	0.39	0.35	0.08	0.20	0.38
PFDA (ng/ml)					
T1 (< 0.3)	183.1 ± 7.9	138.9 ± 11.3	89.0 ± 5.3	7.9 ± 0.4	14.5 ± 0.9
T2 (0.3–0.5)	186.6 ± 7.5	119.7 ± 10.7	88.2 ± 5.0	8.1 ± 0.4	14.3 ± 0.9
T3 (> 0.5)	192.1 ± 7.6	129.3 ± 10.8	95.6 ± 5.0	8.5 ± 0.4	15.0 ± 0.9
*P*_trend_	0.17	0.32	0.13	0.12	0.52

Data are least squares (LS) means ± standard error (SE). ^a^Values were adjusted for age (years), sex (men, women), race (White, non-White), educational attainment (high school or less, some college, and college graduate or beyond), smoking status (never, former, and current smoker), alcohol consumption (drinks/week), physical activity (MET-hr/wk), BMI (kg/m^2^), regular lipid-lowering medication use (yes or no), and dietary intervention groups (categorical)

**Fig. 1:**
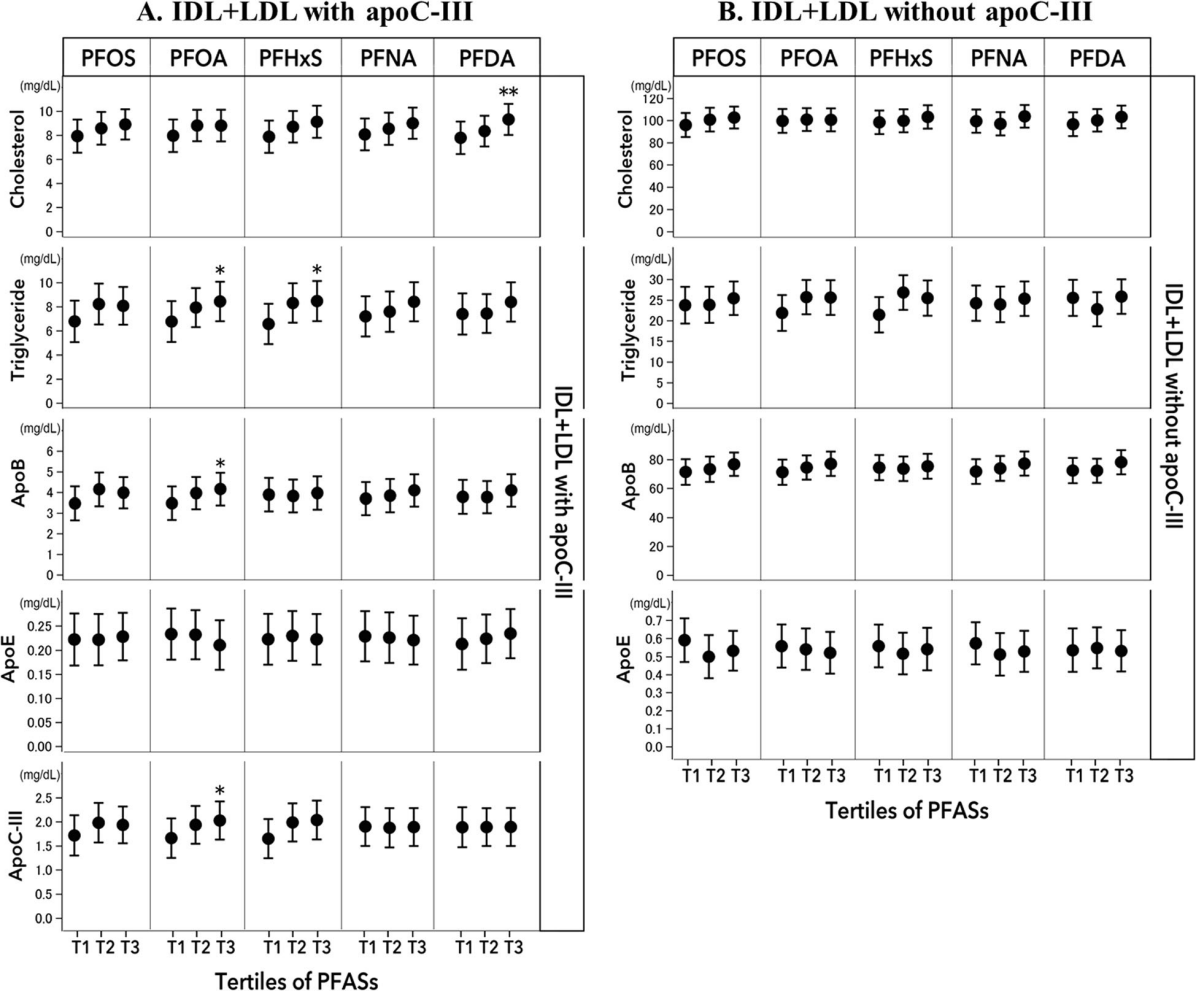
Least-square means of baseline lipids and apolipoproteins in IDL and LDL fraction that contains (**a**) and lacks apoC-III (**b**) according to tertile of PFAS. Error bars indicate the standard error. Values were adjusted for age (years), sex (men, women), race (white, non-white), educational attainment (high school or less, some college, and college graduate or beyond), smoking status (never, former, and current smoker), alcohol consumption (drinks/week), physical activity (MET-hr/wk), BMI (kg/m^2^), regular lipid-lowering medication use (yes or no), and dietary intervention groups (categorical). * *P* trend< 0.05; ** *P* trend < 0.01

Figure 1 shows the associations between PFASs and lipid and apolipoprotein subspecies in IDL+ LDL that contains or lacks apoC-III. After multivariate adjustment, PFAS were primarily associated with lipids/apolipoproteins in IDL and LDL fraction that contains apoC-III. Comparing the highest and lowest tertiles of PFOA, the least-square means (SE) (mg/dl) were 4.16 (0.4) vs 3.47 (0.4) for apoB (*P* trend = 0.04), 2.03 (0.2) vs 1.66 (0.2) for apoC-III (*P* trend = 0.04), and 8.4 (0.8) vs 6.8 (0.8) for triglycerides (*P* trend = 0.03) in IDL and LDL fraction that contains apoCIII (Fig. 1). Significant results were also observed when PFOA concentrations were treated as continuous variables; for each unit increment of log_10_-transformed PFOA, there was a 0.15 mg/dl increment in triglycerides (*P* = 0.03), 0.22 mg/dl increment of apoB (*P* = 0.01), and 0.24 mg/dl increment in apoC-III (*P* = 0.007). In addition, we also found some positive associations for other PFASs. Comparing the highest vs lowest tertiles, the least-square means (SE) (mg/dl) of triglycerides were 8.5 (0.8) vs 6.6 (0.8) for PFHxS (*P* trend = 0.03), the least-square means (SE) of cholesterol were 9.4 (0.6) vs 7.8 (0.7) for PFDA (*P* trend = 0.007). In contrast, no significant association was observed between PFASs and lipids and apolipoproteins in IDL and LDL fraction that lacks apoC-III (Fig. 1).

**Fig. 2:**
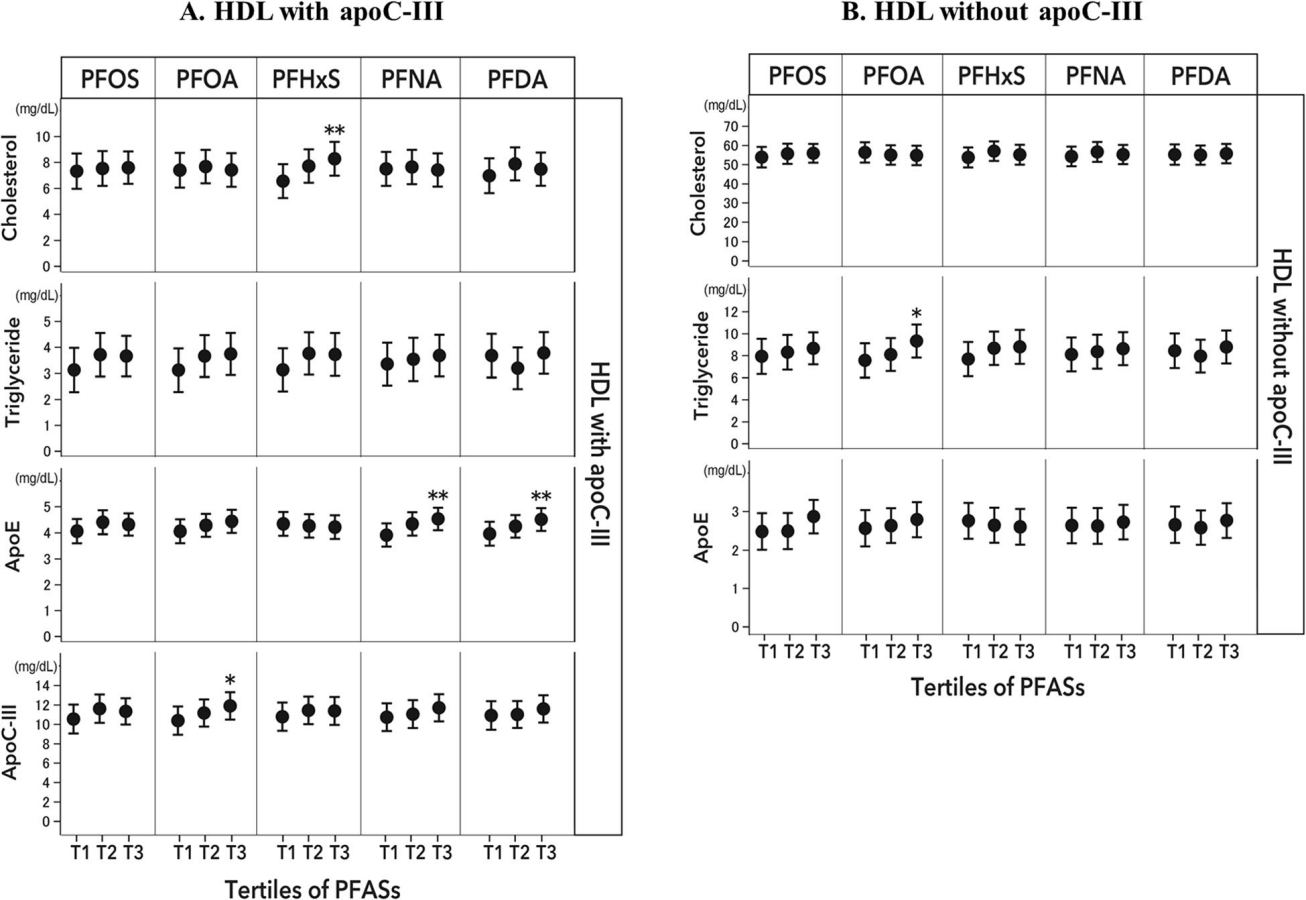
Least-square means of baseline lipids and apolipoproteins in HDL that contains (**a**) and lacks apoC-III (**b**) according to tertile of PFAS. Error bars indicate the standard error. Values were adjusted for age (years), sex (men, women), race (white, non-white), educational attainment (high school or less, some college, and college graduate or beyond), smoking status (never, former, and current smoker), alcohol consumption (drinks/week), physical activity (MET-hr/wk), BMI (kg/m^2^), regular lipid-lowering medication use (yes or no), and dietary intervention groups (categorical). * *P* trend< 0.05; ** *P* trend < 0.01

The associations between PFASs and lipids and apolipoproteins in HDL that contains or lacks apoC-III are shown in Fig. 2. Similar to findings regarding IDL and LDL subspecies, after multivariate adjustment, positive associations with PFASs were primarily observed in HDL that contains apoC-III. Comparing the highest vs lowest tertiles, the least-square means (SE) (mg/dl) of apoC-III were 11.9 (0.7) vs 10.4 (0.7) for PFOA (*P* trend = 0.01). Significant positive associations were also observed between both PFNA and PFDA and apoE, as well as between PFHxS and cholesterol concentrations. Comparing extreme tertiles, the least-square means (SE) of apoE were 4.53 (0.2) vs 3.91 (0.2) for PFNA (*P* trend = 0.004) and 4.51 (0.2) vs 3.96 (0.2) for PFDA (*P* trend = 0.005), and these figures were 8.3 (0.7) vs 6.5 (0.7) for cholesterol concentrations by PFHxS tertiles (*P* trend = 0.008). Consistent results were observed when PFAS concentrations were treated as continuous variables; for each unit increment of log_10_-transformed PFAS, there was a 0.14 mg/dl increment in apoC-III for PFOA (*P* = 0.009), a 0.11 mg/dl increment in apoE for PFNA (*P* = 0.002), and a 0.12 mg/dl increment in apoE for PFDA (*P* = 0.005) (Fig. 2). In contrast, a significant association was only observed between PFOA and triglycerides in HDL that lacks apoC-III (9.3 [0.8] vs 7.6 [0.8], *P* < 0.05).

The associations between PFASs and lipids and apolipoprotein subspecies in VLDL are shown in Additional file 1: Table S2. PFOA and PFOS were positively associated with apoB in VLDL that lacks apoC-III. In addition, PFOS concentrations were inversely associated with concentrations of apoE and cholesterol in VLDL that contains apoC-III, and PFNA was inversely associated with apoB, apoC-III, apoE, and cholesterol contents in VLDL that contains apoC-III.

Additional file 1: Table S3 shows the associations of PFASs with lipoprotein subspecies in men and women. In both genders, PFAS were positively associated with lipids or apolipoproteins in IDL + LDL and HDL that contain apoC-III, although stronger associations that contains apoC-III concentrations were observed in women. A similar pattern of associations was observed in race-stratified analyses, although most of the associations did not reach statistical significance among non-whites largely due to limited power (*n* = 48; data not shown). In addition, the results did not significantly change when menopausal status and hormone replacement therapy use were further adjusted, or when analysis was restricted to participants without lipid-lowering medication use or non-current smokers (data not shown). Similar patterns of associations were demonstrated between baseline PFAS concentrations and lipoprotein subspecies at 2 years after the intervention (Additional file 1: Table S4). PFASs were not associated with changes in lipoprotein subspecies from baseline to 2 years. No significant interaction was observed between dietary intervention groups and PFAS exposure on levels of lipoprotein subspecies.

## Discussion

In this 2-year randomized trial in overweight and obese participants, we found that PFAS exposure levels were positively associated with plasma concentrations of apoB and apoC-III, but not with total cholesterol or triglycerides. Furthermore, higher PFAS concentrations were primarily associated with higher concentrations of cholesterol, triglycerides, and apolipoproteins in IDL, LDL, and HDL that contain apoC-III. These associations were independent of potential confounders including diet and lipid-lowering medication use and somewhat stronger among women. In addition, similar associations were observed when we examined baseline PFAS concentrations in relation to lipoprotein subspecies by the end of trial, thus suggesting that these findings were robust to changes in body weight during the trial.

Most animal studies showed that administration of PFAS led to lower levels of circulating lipids (i.e., total cholesterol and triglycerides), but known between-species differences in PFAS toxicokinetics limit the generalizability of animal study findings to humans [1]. Evidence from human studies regarding PFAS exposure and lipids is mixed [11, 27, 28]. Positive associations between PFASs and total cholesterol, LDL cholesterol, or triglycerides were observed in some cross-sectional and prospective studies [4, 8, 9, 29–31], whereas other studies reported null associations or even inverse associations [11, 28, 32]. We are among the first to examine apolipoprotein species within the broad categories of lipoproteins that were not considered in previous studies [13, 14]. Indeed, the present study showed no clear association between PFAS and total cholesterol, triglycerides, or lipoproteins in plasma, but PFAS exposures were associated with apoC-III levels and also the lipid contents in IDL, LDL, and HDL particles that contain apoC-III. The diverging functions of apolipoproteins in the same class of blood lipids may also explain the previous inconsistent findings regarding PFAS exposures and total blood lipid levels.

Current evidence suggests that blood lipid particles are highly heterogeneous and comprise a group of lipoproteins and apolipoproteins (such as apoC and apoE) with diverse biological functions [13, 15–17]. For instance, studies have shown that LDL that contains apoC-III, but not LDL that lacks apoC-III, was an independent risk factor for cardiovascular risk [18–20]. Two independent prospective studies showed that HDL cholesterol that contains or lacks apoC-III demonstrated opposite associations with the risk of coronary heart disease (CHD): HDL cholesterol that lacks apoC-III was inversely associated with CHD, whereas HDL cholesterol that contains apoC-III (small subfraction) was associated with a higher risk of CHD [16]. Further, the associations of apoE concentrations in HDL with cardiovascular risk significantly differ in the presence of apoC-III in that HDL with both apoE and apoC-III tended to be associated with a higher cardiometabolic risk [17, 33, 34]. Therefore, the heterogeneous lipoprotein subspecies deserve to be characterized in order to improve the disease risk prediction rather than relying on total lipid fractions [14]. Our finding of PFAS exposures being primarily associated with IDL, LDL, and HDL subfractions containing apoC-III suggests that elevated PFAS exposure may potentially exert increased cardiovascular risk [19], as already suggested by recent evidence [35]. We estimated that the difference in apoC-III levels between the extreme tertiles of PFOA would lead to an 18% increased risk of cardiovascular disease (CVD), based on a pooled estimate of 148% increased CVD risk for each 5-mg/dl increase in apoC-III levels [20].

The mechanisms underlying our findings are not well understood. Evidence from animal studies has suggested that the biological effects of PFASs might be attributed to the activation of PPAR-α [3], but PFOA may also alter the expression of proteins regulated by hepatocyte nuclear factor 4α [36], a key regulator of lipid metabolism [37]. However, these findings may not be extrapolated to humans. As another possibility, in vitro studies report that PFASs may bind to phospholipid membranes, thereby influencing membrane fluidity [38]. Thus, given the long half-life of the PFASs in human blood [1], accumulation in membranes might well cause long-term adverse effects on cell functions including lipid metabolism. Of note, we cannot exclude the possibility that our observed associations may not bear any causal interpretation if PFASs are incorporated in the same lipoprotein species that contain apoC-III, although we believe that such possibility is small because the current evidence suggests that the majority of PFASs in circulation are carried by albumin rather than lipoproteins [39]. In addition, in a sensitivity analysis, we observed similar results when we examined the ratio of IDL, LDL, and HDL particles that contain apoC-III to total cholesterol levels as a surrogate measure of lipoprotein compositions. More studies are warranted to elucidate the distribution of PFASs in blood compartments and other tissues in human body.

To our knowledge, this is among the first studies to investigate the associations between PFAS exposure and lipoprotein and apolipoprotein subspecies in adults. The present study accounted for a multitude of potential confounding factors, including diet and lifestyle factors, BMI, and lipid-lowering medication use. However, our study is also subject to some limitations. First, our primary findings were based on a cross-sectional analysis, although similar patterns of associations were observed between baseline PFAS concentrations and lipoprotein and apolipoproteins measured 2 years later. Second, our study participants were overweight or obese and had relatively homogeneous health status, and therefore our findings may not be extrapolated to populations with other characteristics. Third, the role of multiple testing must be considered, although we chose not to apply the conservative Bonferroni correction in the analyses given the inter-correlation between the PFASs (*r*_s_ ranged from 0.4 to 0.9). Fourth, we measured only baseline plasma PFAS concentrations and could not examine the associations of changes in PFASs and lipoprotein subspecies during the follow-up. Fifth, the associations tended to be more pronounced in women, but we do not have sufficient statistical power to formally test potential gender or racial differences. Finally, residual or unmeasured confounding could not be entirely ruled out in this observational study.

## Conclusions

Plasma PFAS levels were not associated with blood lipids in overweight or obese individuals who participated in a clinical trial, although plasma levels of PFOA were significantly associated with higher apoC-III, as well as levels of cholesterol, triglycerides, and other apolipoproteins in subspecies of IDL, LDL, and HDL that contain apoC-III. These novel findings suggest that PFAS exposures might interfere with lipid metabolism not reflected by routine lipid parameters and that the potential detrimental effects of PFASs on promoting atherogenic lipid subfractions may potentially lead to an elevated risk of developing CVD.

## Supplementary information

**Additional file 1: Figure S1.** Heatmap of correlations between lipoprotein and apolipoprotein subspecies*. **Table S1.** Comparisons of characteristics between included and excluded participants. **Table S2.** Partial Spearman correlation coefficients between baseline PFASs and lipids and apolipoproteins in VLDL. **Table S3.** Partial Spearman correlation coefficients between PFASs and lipoproteins and apolipoproteins in men and women. **Table S4.** Partial Spearman correlation coefficients between baseline PFASs and lipoproteins and apolipoproteins at 24th month after the diet intervention.

## Data Availability

The data supporting the conclusions of this work are included within the manuscript and its Additional files. Further, the dataset analyzed during the current study is available from the corresponding author on reasonable request.
