# Supplementary material for: Associations of Perfluoroalkyl substances with blood lipids and Apolipoproteins in lipoprotein subspecies: the POUNDS-lost study
Source: Environ Health. 2020 Jan 13;19:5. doi: 10.1186/s12940-020-0561-8 (PMC6958662; doi:10.1186/s12940-020-0561-8)
Supplement: Supplementary file 1 — Additional file 1: Figure S1. Heatmap of correlations between lipoprotein and apolipoprotein subspecies*. Table S1. Comparisons of characteristics between included and excluded participants. Table S2. Partial Spearman correlation coefficients between baseline PFASs and lipids and apolipoproteins in VLDL. Table S3. Partial Spearman correlation coefficients between PFASs and lipoproteins and apolipoproteins in men and women. Table S4. Partial Spearman correlation coefficients between baseline PFASs and lipoproteins and apolipoproteins at 24th month after the diet intervention. [file 12940_2020_561_MOESM1_ESM.docx]

**Figure S1.** Heatmap of correlations between lipoprotein and apolipoprotein subspecies*

***** Values were partial Spearman correlation coefficients, adjusting for age, sex, race, education, smoking status, alcohol consumption, physical activity, body mass index, regular lipid-lowering medication use, and dietary intervention groups.

**Table S1**. Comparisons of characteristics between included and excluded participants

|  | **Included (n=326)** | **Non-included (n=485)** | ***P* value** |
| --- | --- | --- | --- |
| Age (years) | 52.7 ± 8.7 | 49.6 ± 9.3 | <0.001 |
| Sex, men, % | 38.9 | 34.9 | 0.23 |
| Race, White, % | 85.3 | 75.3 | 0.01 |
| BMI (kg/m^2^) | 32.3 ± 3.8 | 32.9 ± 3.9 | 0.08 |
| Body weight (kg) | 91.7 ± 15.3 | 93.7 ± 15.6 | 0.10 |
| Education level, high school or less, % | 9.8 | 9.1 | 0.10 |
| Current smoker, yes, % | 3.5 | 4.7 | 0.72 |
| Alcohol consumption (drinks/week) | 2.1 ± 2.8 | 2.2 ± 3.0 | 0.65 |
| Physical activity* | 1.58 ± 0.1 | 1.58 ± 0.1 | 0.68 |
| Systolic blood pressure (mmHg) | 119.8 ± 13.1 | 119.2 ± 13.6 | 0.57 |
| Diastolic blood pressure (mmHg) | 75.1 ± 8.9 | 75.5 ± 9.7 | 0.53 |

*Physical activity was estimated by the Baecke Questionnaire.

**Table S2.** Partial Spearman correlation coefficients between baseline PFASs and lipids and apolipoproteins in VLDL

|  | **PFOS** | **PFOA** | **PFHxS** | **PFNA** | **PFDA** |
| --- | --- | --- | --- | --- | --- |
| **VLDL containing ApoC-III** | | | | | |
| ApoB | -0.11 | -0.05 | -0.05 | -0.14* | -0.10 |
| ApoC-III | -0.08 | -0.01 | 0.001 | -0.14* | -0.09 |
| ApoE | -0.13* | -0.07 | -0.06 | -0.18* | -0.09 |
| Triglycerides | -0.06 | -0.02 | 0.004 | -0.09 | -0.07 |
| Cholesterol | -0.13* | -0.09 | -0.06 | -0.16* | -0.09 |
| **VLDL lacking ApoC-III** | | | | | |
| ApoB | 0.11* | 0.14* | 0.09 | 0.08 | 0.07 |
| ApoE | -0.09 | 0.02 | -0.03 | -0.08 | -0.05 |
| Triglycerides | 0.01 | 0.05 | 0.02 | -0.04 | -0.05 |
| Cholesterol | 0.06 | 0.07 | 0.03 | 0.03 | 0.03 |

Values were adjusted for age, sex, race, education, smoking status, alcohol consumption, physical activity, body mass index, regular lipid-lowering medication use, and dietary intervention groups.

* *P*<0.05; ***P*<0.01

**Table S3.** Partial Spearman correlation coefficients between PFASs and lipoproteins and apolipoproteins in men and women

|  | **PFOS** | **PFOA** | **PFHxS** | **PFNA** | **PFDA** |
| --- | --- | --- | --- | --- | --- |
| **Men (n=127)** | | | | | |
| **IDL+LDL containing ApoC-III** | | | | | |
| ApoB | 0.06 | 0.15 | -0.02 | -0.01 | 0.03 |
| ApoC-III | -0.12 | 0.05 | -0.05 | -0.16 | -0.13 |
| ApoE | -0.05 | -0.03 | -0.08 | -0.01 | 0.02 |
| Triglycerides | 0.09 | 0.17* | 0.18* | 0.01 | -0.02 |
| Cholesterol | 0.06 | 0.11 | 0.09 | -0.03 | 0.05 |
| **HDL containing ApoC-III** | | | | | |
| ApoC-III | -0.03 | 0.05 | -0.03 | -0.17 | -0.09 |
| ApoE | 0.05 | 0.18* | 0.07 | 0.08 | 0.13 |
| Triglycerides | 0.16 | 0.15 | 0.20* | 0.08 | 0.06 |
| Cholesterol | 0.19* | 0.22* | 0.15 | 0.07 | 0.11 |
| **Women (n=199)** | | | | | |
| **IDL+LDL containing ApoC-III** | | | | | |
| ApoB | 0.14* | 0.07 | 0.09 | 0.21** | 0.17* |
| ApoC-III | 0.14* | 0.14* | 0.14* | 0.15* | 0.12 |
| ApoE | 0.05 | -0.04 | 0.05 | -0.02 | 0.07 |
| Triglycerides | 0.16* | 0.08 | 0.14 | 0.18** | 0.18** |
| Cholesterol | 0.15* | 0.05 | 0.06 | 0.17* | 0.20** |
| \| **HDL containing ApoC-III** \| \| --- \| | | | | | |
| ApoC-III | 0.19** | 0.18** | 0.15* | 0.28*** | 0.15* |
| ApoE | 0.12 | 0.06 | -0.10 | 0.23** | 0.20** |
| Triglycerides | 0.13 | 0.05 | 0.13 | 0.16* | 0.14 |
| Cholesterol | 0.12 | -0.02 | 0.10 | 0.08 | 0.13 |

Values were adjusted for age, race, education, smoking status, alcohol consumption, physical activity, body mass index, regular lipid-lowering medication use, and dietary intervention groups.

* *P*<0.05; ***P*<0.01; ****P*<0.001

**Table S4.** Partial Spearman correlation coefficients between baseline PFASs and lipoproteins and apolipoproteins at 24^th^ month after the diet intervention

|  | **PFOS** | **PFOA** | **PFHxS** | **PFNA** | **PFDA** |
| --- | --- | --- | --- | --- | --- |
| **IDL+LDL containing ApoC-III** | | | | | |
| ApoB | 0.09 | 0.11* | 0.07 | 0.06 | 0.01 |
| ApoC-III | 0.05 | 0.13* | 0.10 | 0.02 | -0.02 |
| ApoE | 0.02 | -0.02 | 0.01 | -0.01 | 0.02 |
| Triglycerides | 0.02 | 0.04 | 0.04 | -0.01 | 0.02 |
| Cholesterol | 0.11* | 0.07 | 0.11* | 0.05 | 0.04 |
| **IDL+LDL lacking ApoC-III** | | | | | |
| ApoB | 0.10 | 0.13* | 0.12 | 0.10 | 0.07 |
| ApoE | -0.02 | 0.02 | 0.06 | -0.004 | -0.01 |
| Triglycerides | -0.03 | 0.01 | 0.07 | -0.04 | -0.001 |
| Cholesterol | 0.07 | 0.05 | 0.10 | 0.03 | 0.03 |
| **HDL containing ApoC-III** | | | | | |
| ApoC-III | 0.05 | 0.01 | -0.03 | 0.02 | 0.003 |
| ApoE | 0.13* | 0.12* | 0.003 | 0.13* | 0.13* |
| Triglycerides | 0.02 | -0.03 | 0.006 | -0.05 | 0.02 |
| Cholesterol | 0.06 | -0.05 | -0.03 | -0.07 | -0.02 |
| \| **HDL lacking ApoC-III** \| \| --- \| | | | | | |
| ApoE | 0.04 | 0.01 | -0.06 | 0.03 | 0.04 |
| Triglycerides | 0.02 | -0.02 | 0.03 | -0.04 | -0.01 |
| Cholesterol | -0.01 | -0.12 | -0.02 | -0.05 | -0.01 |

Values were adjusted for age, sex, race, education, smoking status, alcohol consumption, physical activity, baseline body mass index, body weight change during 0-6 months and 6-24 months, regular lipid-lowering medication use, and dietary intervention groups.

* *P*<0.05; ***P*<0.01
